# Supplementary material for: Transcriptome Analysis Reveals HgCl2 Induces Apoptotic Cell Death in Human Lung Carcinoma H1299 Cells through Caspase-3-Independent Pathway
Source: Int J Mol Sci. 2021 Feb 18;22(4):2006. doi: 10.3390/ijms22042006 (PMC7922270; doi:10.3390/ijms22042006)

Supplementary Figure 1

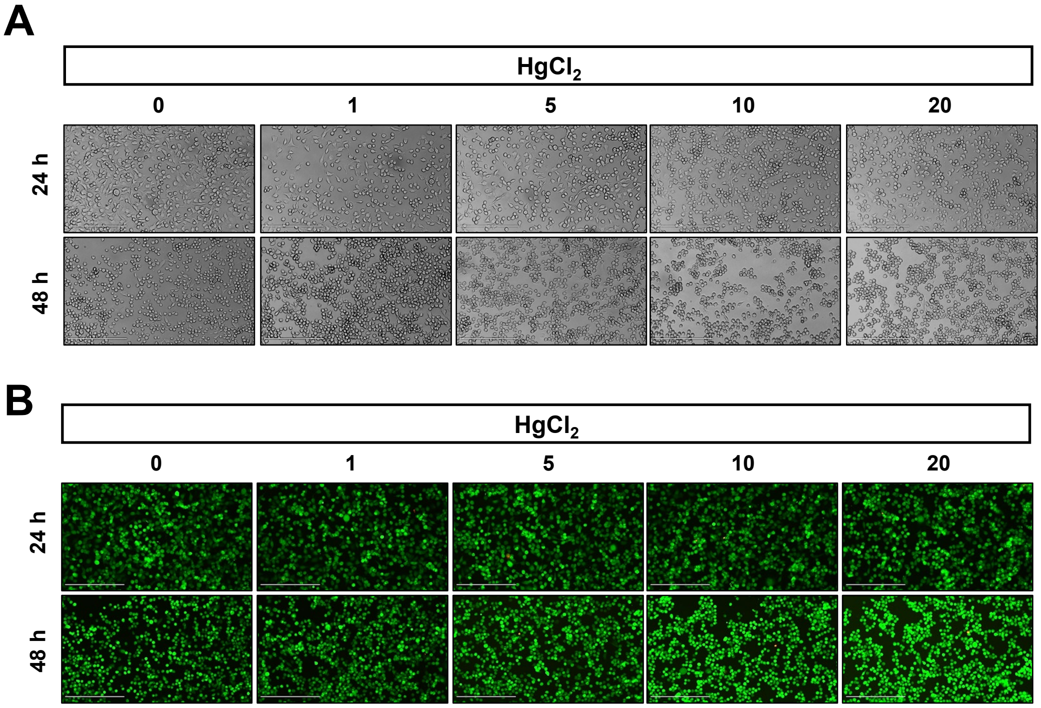

Supplementary Figure 2

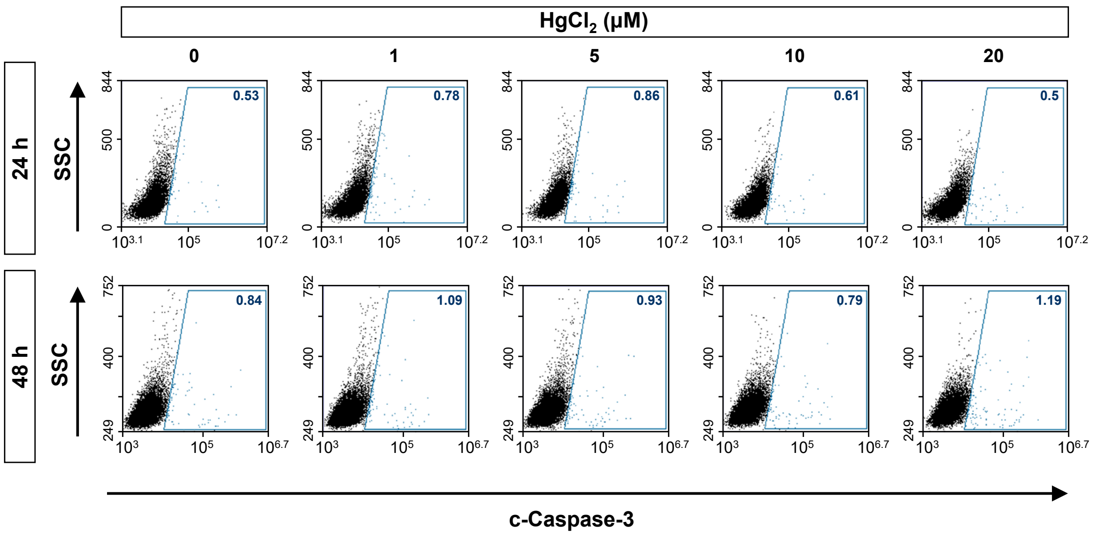

Supplementary Figure 3

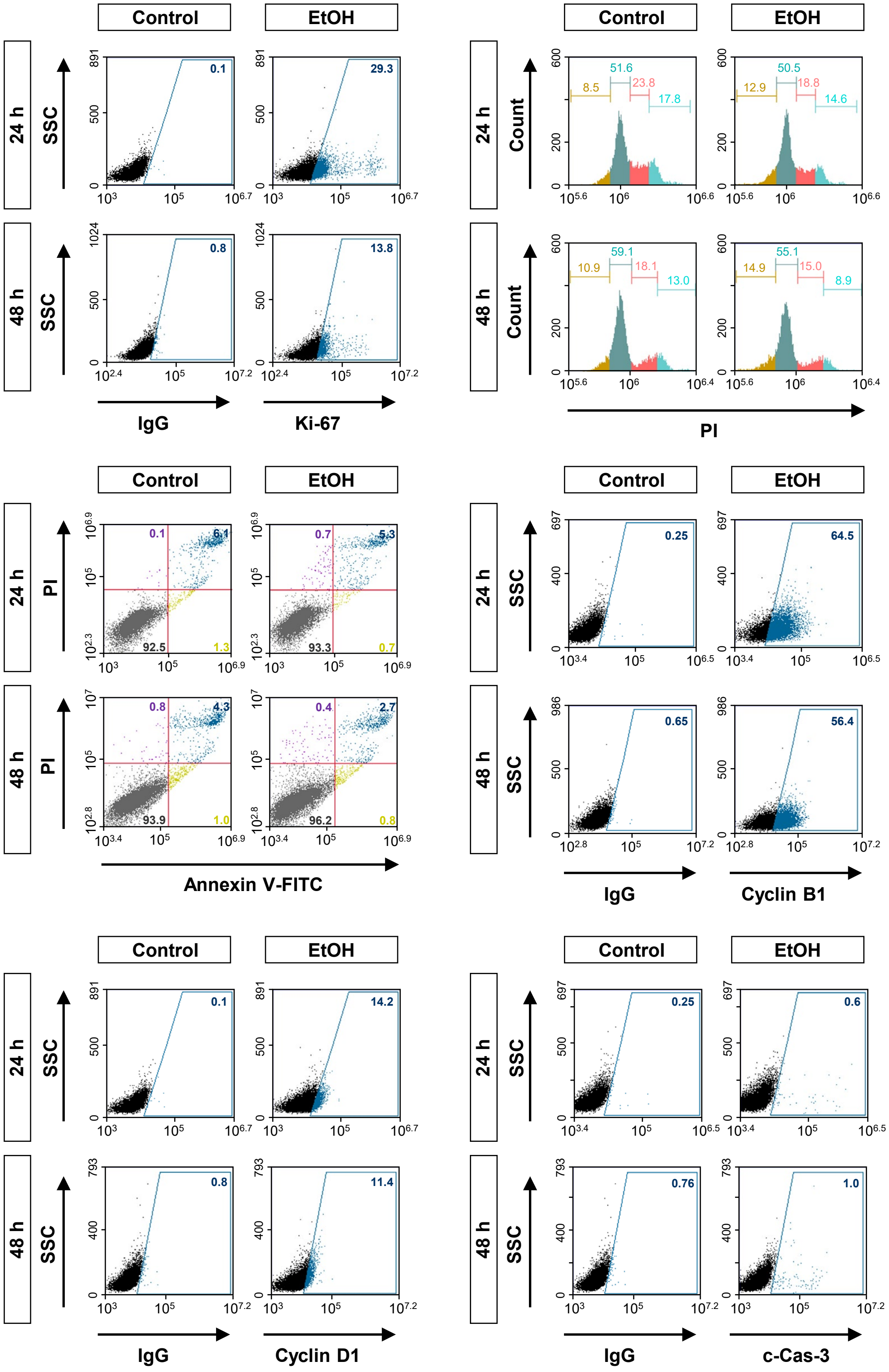

Supplementary Figure 4

A

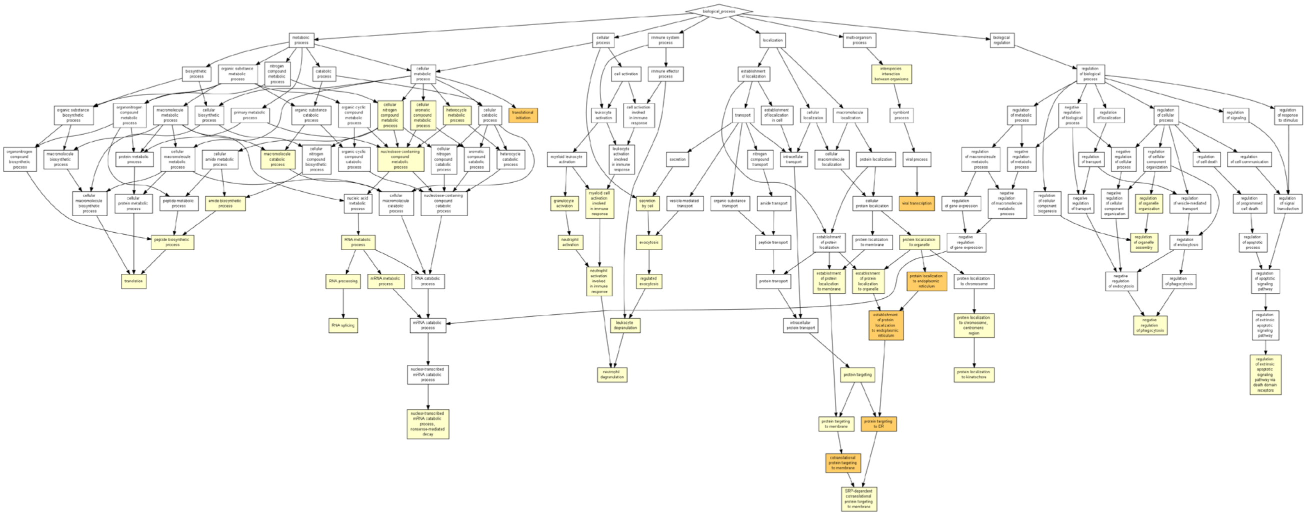

B

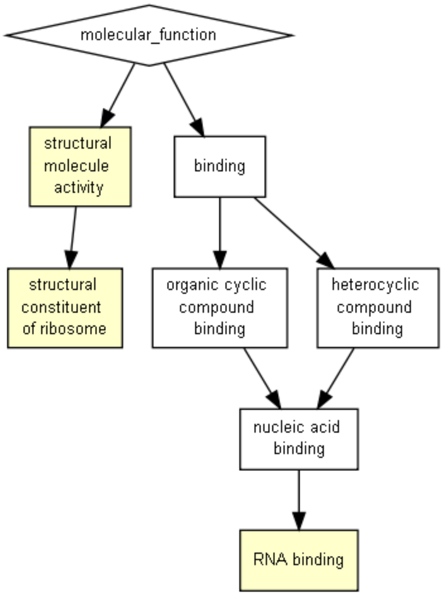

Supplementary Figure 5

A

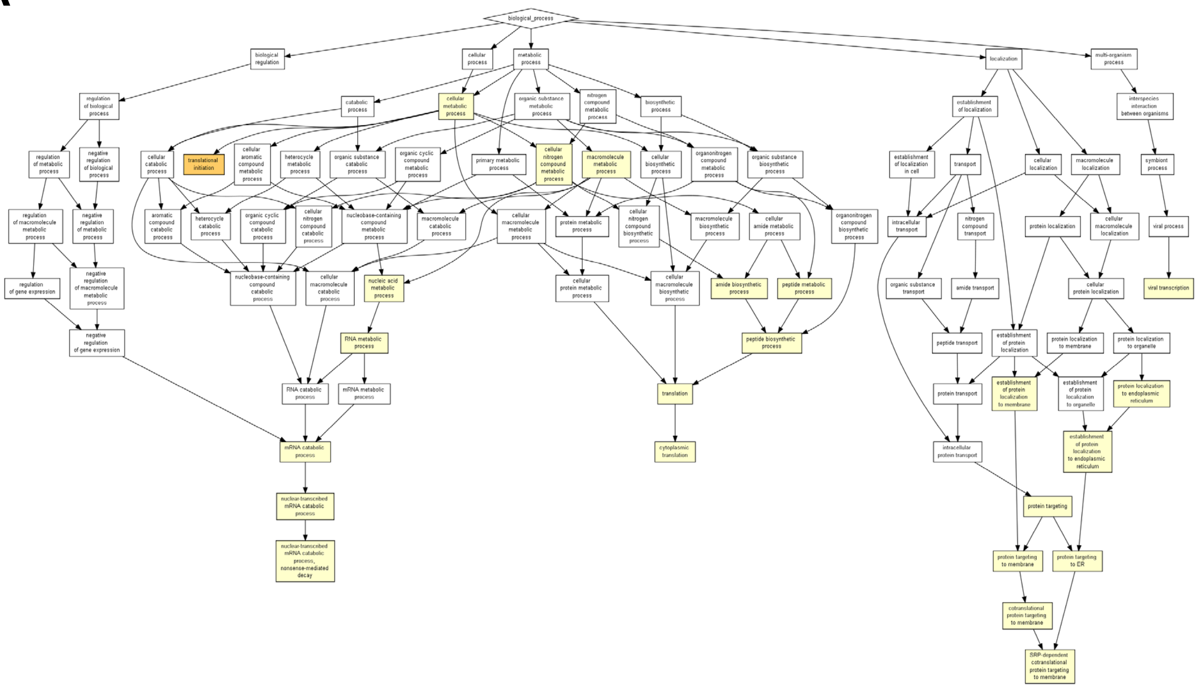

B

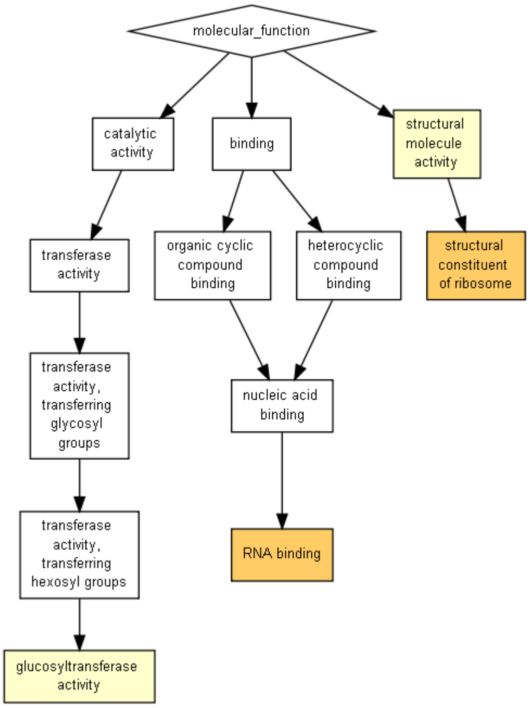

Supplement: Supplementary file 1 [file ijms-22-02006-s001.zip › Supplementary Figures.pdf]
