# Supplementary material for: Transcriptome Analysis Reveals HgCl2 Induces Apoptotic Cell Death in Human Lung Carcinoma H1299 Cells through Caspase-3-Independent Pathway
Source: Int J Mol Sci. 2021 Feb 18;22(4):2006. doi: 10.3390/ijms22042006 (PMC7922270; doi:10.3390/ijms22042006)
Supplement: Supplementary file 1 [file ijms-22-02006-s001.zip › Supplementary Material.docx]

Supplementary Material


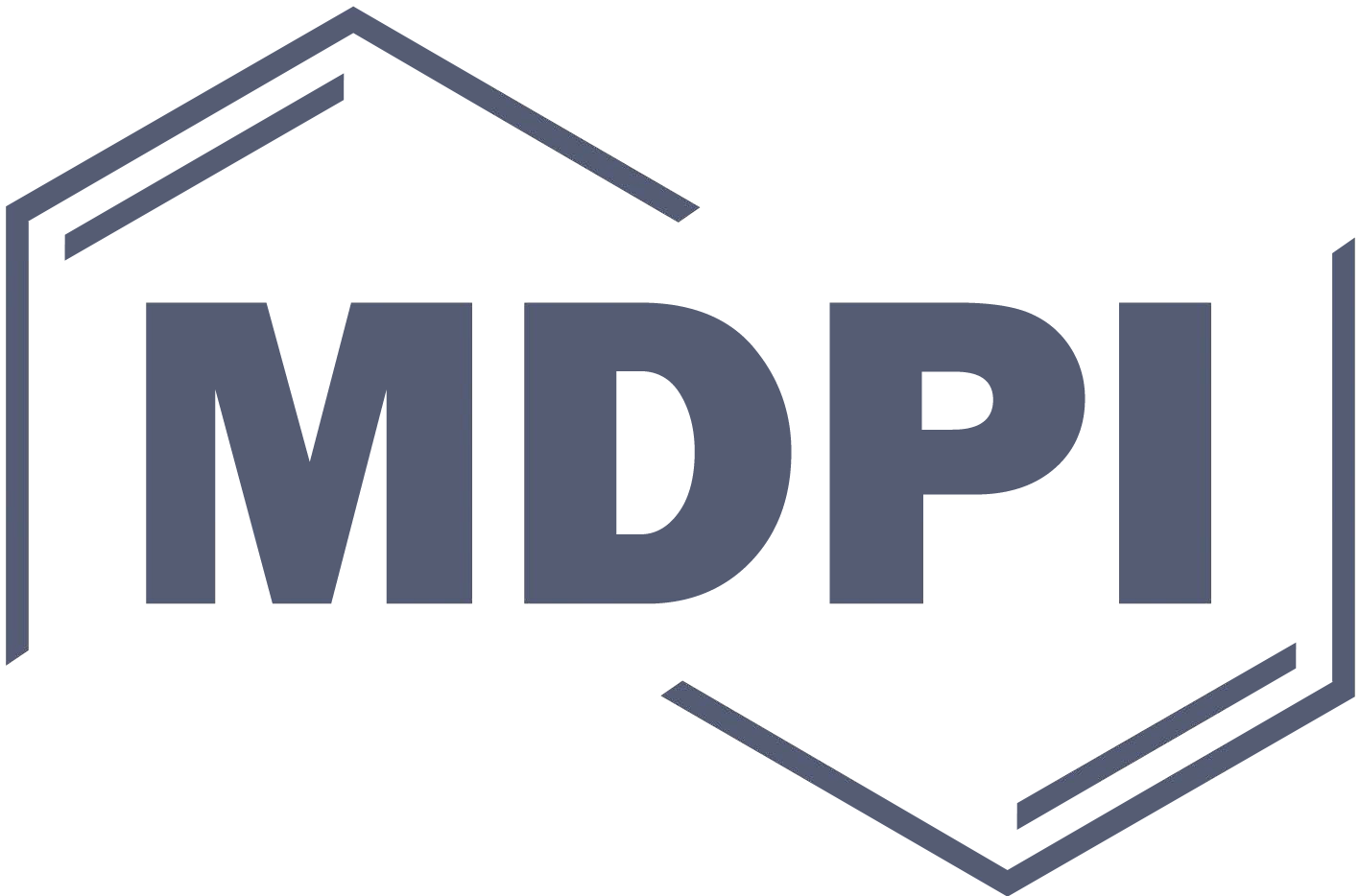


Transcriptome analysis reveals mercury chloride induces apoptotic cell death in human lung carcinoma cells, H1299 through caspase-3-independent pathway

Mi Jin Kim^1^, Jinhong Park^1^, Jin-Ho Kim^1^, Ji-Young Kim^1^, Mi-Jin An^1^, Geun-Seup Shin^1^, Hyun-Min Lee^1^, Chul-Hong Kim^1^, and Jung-Woong Kim^1,*^

^1^ Department of Life Science, Chung-Ang University, Seoul 06974, South Korea;

***** Correspondence: jungkim@cau.ac.kr; Tel.: +82-2-820-6682, Fax: +82-2-815-6682

**Figure S1.** Effect of mercury chloride on cell viability and morphologic changes of H1299 cells. (**A**) Cells were treated with different concentrations (0-20 μM) for 24 or 48 h. The morphology of H1299 cells was observed by phase-contrast microscopy. (**B**) H1299 cells were stained with calcein-AM (green) and ethidium homodimer (red) by the live/dead assay. 0.6% of EtOH were used as the negative control. Images are representative of three independent experiments. Scale bars represent 200 μm.


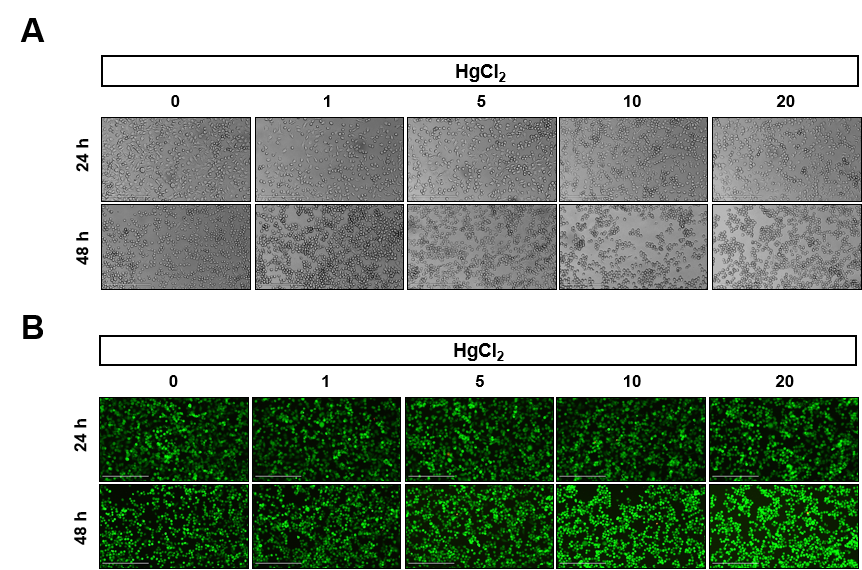

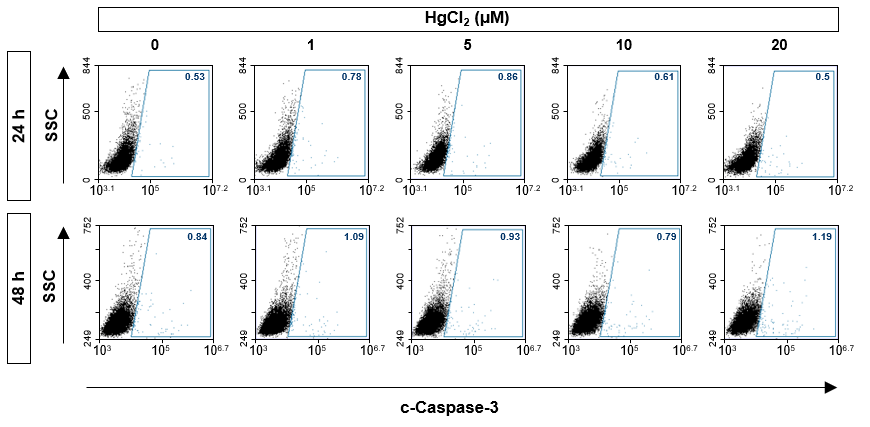


**Figure S2.** The effect of caspase-3 activity in HgCl_2_-treated H1299 cells. (**A**) After treatment of HgCl_2_ for 24 or 48 h. Cells were fixed with 1 % PFA for 6 hrs and stained with anti-cleaved caspase-3 antibody. Expression levels of cleaved caspase-3 were analyzed by FACS analysis. Data are represented as the mean ± S.E.M. of three independent experiments (n = 6), each performed in triplicate. 0.6% EtOH was used as the negative control.


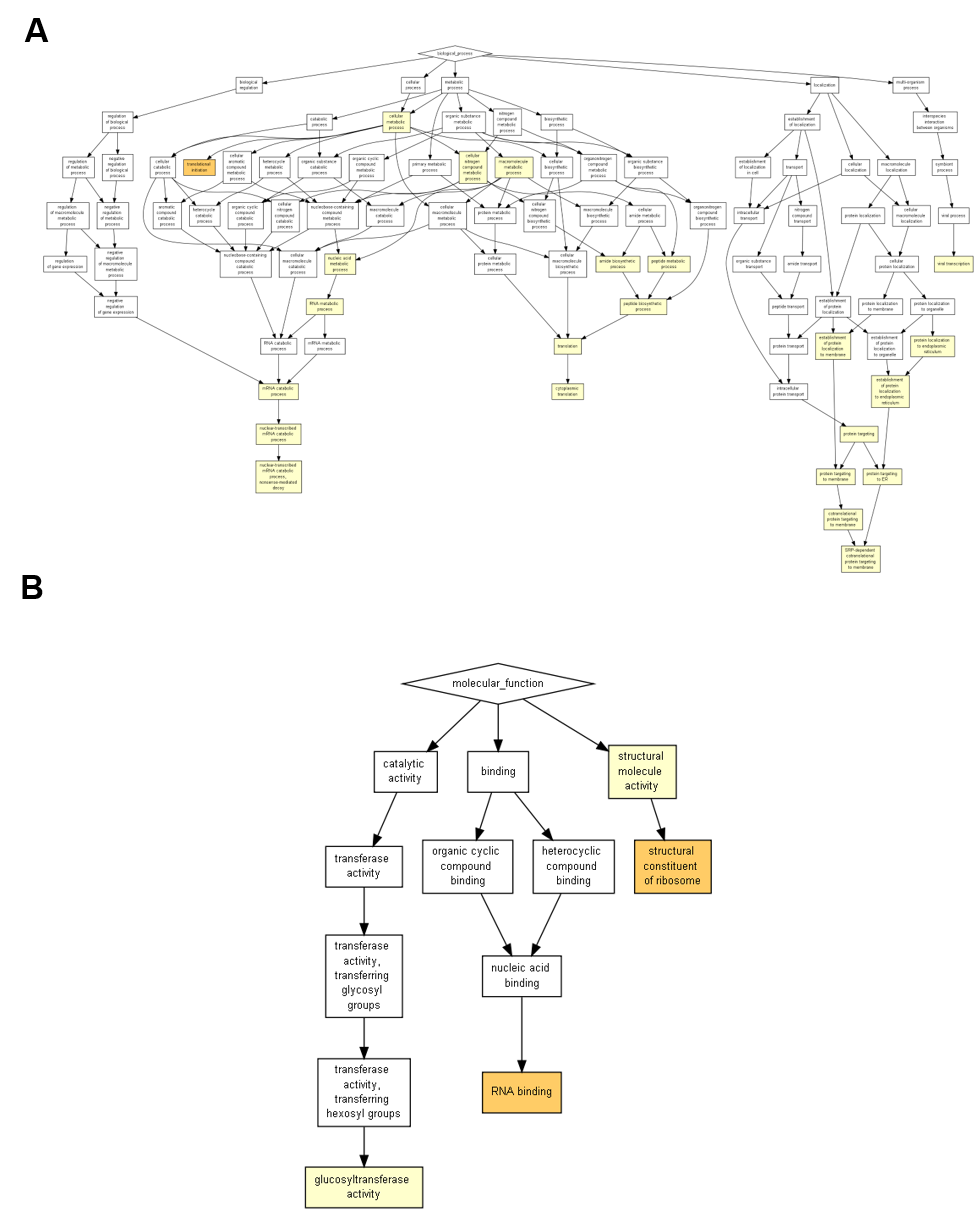


**Figure S3.** A visualization of the biological process Gene Ontology (GO) annotations using Gorilla tool. Cells were treated 5 μM HgCl_2_ for 48 h, and then the construction of RNA-sequencing library was performed using 1 μg of isolated total RNA. The dataset for upregulated genes of transcriptome profiling show enriched GO terms of biological processes (A) and molecular function (B). The color indicates the degree of enrichment, from dark orange which means significantly enriched, to white which means not enriched. All was selected for ontogeny choices and the P-value < 0.01.


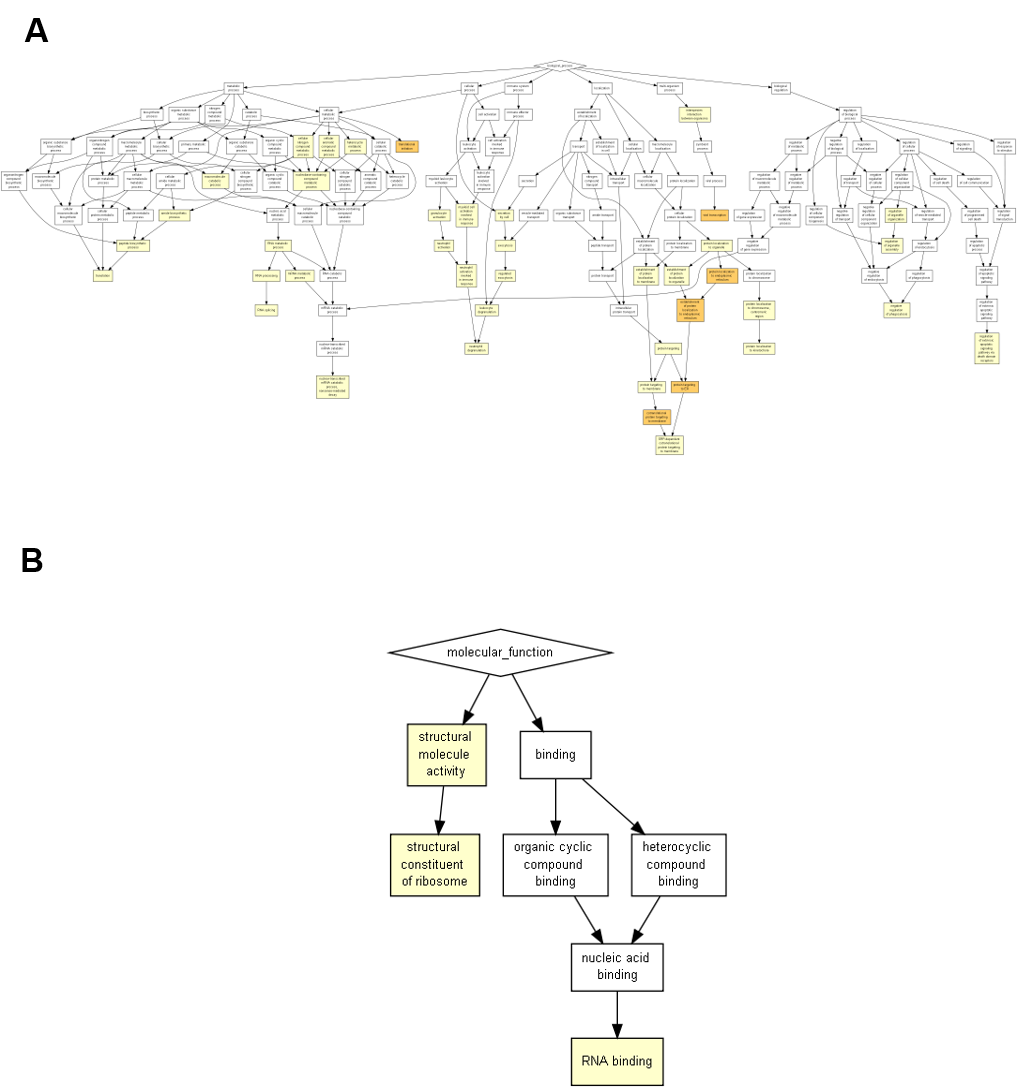
 **Figure S4.** A visualization of the biological process Gene Ontology (GO) annotations using Gorilla tool. The dataset for downregulated genes of transcriptome profiling show enriched GO terms of biological processes (A) and molecular function (B). The color indicates the degree of enrichment, from dark orange which means significantly enriched, to white which means not enriched. All was selected for ontogeny choices and the P-value < 0.01.

**Publisher’s Note:** MDPI stays neutral with regard to jurisdictional claims in published maps and institutional affiliations.

| 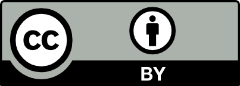 | © 2020 by the authors. Submitted for possible open access publication under the terms and conditions of the Creative Commons Attribution (CC BY) license (http://creativecommons.org/licenses/by/4.0/). |
| --- | --- |
